# Evaluation of administration-related reactions with subcutaneous daratumumab with and without premedication

**DOI:** 10.1093/oncolo/oyae158

**Published:** 2024-06-26

**Authors:** Kashyap Padmaraju, Kaitlin Kelly, Andrzej J Jakubowiak, Benjamin A Derman

**Affiliations:** Department of Pharmacy, University of Chicago Medical Center, Chicago, IL, United States; Department of Pharmacy, University of Chicago Medical Center, Chicago, IL, United States; Section of Hematology/Oncology, Department of Medicine, University of Chicago, Chicago, IL, United States; Section of Hematology/Oncology, Department of Medicine, University of Chicago, Chicago, IL, United States

**Keywords:** multiple myeloma, amyloidosis, daratumumab

## Abstract

**Background:**

Daratumumab-hyaluronidase-fihj (Dara-SQ) is frequently used in the treatment of plasma cell disorders and is associated with improved outcomes. Dara-SQ was shown to be non-inferior to intravenous daratumumab (Dara-IV) in efficacy, safety, and associated with fewer administration-related reactions (ARRs). Despite the lower ARR risk with Dara-SQ, package labeling still recommends indefinite premedication. In this study, we investigated the safety of premedication discontinuation after one cycle of Dara-SQ.

**Materials and Methods:**

This pre-post interventional quality improvement study included all patients aged 18 years and older diagnosed with multiple myeloma or light chain (AL) amyloidosis who received at least one dose of Dara-SQ. Patients in Arm 1 received Dara-SQ per package labeling, while patients in Arm 2 had premedication omitted (excluding dexamethasone) after cycle 1. The primary endpoint was the incidence of ARR after cycle 1. Overall ARR rate and therapy time saved were also evaluated.

**Results:**

A total of 102 patients (63 in Arm 1 and 39 in Arm 2) were included. There were zero reactions in either arm after cycle 1 across 1479 Dara-SQ doses administered over a 30-month period with or without premedication omission. The overall ARR rate was 2.9% (3/102), which all occurred prior to premedication omission. Therapy timed saved from premedication omission was 194 hours in a 6-month period, equating to approximately $140 000 USD.

**Conclusion:**

ARRs to Dara-SQ were rare, mild, and occurred during cycle 1 prior to premedication omission. Omission of noncorticosteroid premedication is safe, feasible, and carries substantial time and cost savings for patients and infusion centers.

## Teaser

Daratumumab-hyaluronidase-fihj (Dara-SQ), used in the treatment of plasma cell disorders, is associated with improved outcomes. This study investigated the safety of premedication discontinuation after one cycle of Dara-SQ.

**Implications for Practice**

Daratumumab-hyaluronidase-fihj (Dara-SQ) administration-related reaction (ARR) risk reduces significantly after the first cycle. Indefinite premedication is recommended per package labeling despite this and may be unnecessary. This quality improvement study assessed the safety of premedication omission after one cycle and its impact on infusion clinic care. Omission of premedication after one cycle was safe and can substantially improve the quality of care delivered for patients and infusion centers. This can be broadly applicable as daratumumab-hyaloridase-fihj is used heavily in the treatment of plasma cell disorders.

## Introduction

Daratumumab is a humanized monoclonal antibody (mAB) that targets CD38 glycoproteins found on the surface of malignant plasma cells and exerts its effects through: antibody-dependent cell-mediated cytotoxicity and induction of apoptosis.^1^ Daratumumab plays a pivotal role in the treatment of plasma cell disorders regardless of transplant intent or disease setting and is associated with improved outcomes when added to standard backbones for multiple myeloma and light chain (AL) amyloidosis.^2,3^

Intravenous daratumumab (Dara-IV) was the first formulation approved and accounted for a high incidence of administration-related reactions (ARRs) during the first dose. In Dara-IV monotherapy and combination clinical trials: *N* = 2066, ARRs occurred with 37% of patients with first dose, 2% with second dose, and cumulatively 6% with subsequent infusions.^4^ This prompted many to adopt a 90-minute infusion protocol after the second dose with indefinite premedication of acetaminophen, diphenhydramine, corticosteroid, and optional montelukast and albuterol.^4,5^ Subcutaneous daratumumab-hyaluronidase-fihj (Dara-SQ) was later shown to be non-inferior to Dara-IV in terms of safety, progression-free survival, and overall survival; only 13% of patients on Dara-SQ experienced an ARR compared to 35% of patients receiving Dara-IV with a median onset time of 3.5 hours (SQ) versus 1.5 hours (IV)^,^.^6^

Dara-SQ is simple to prepare and administer while reducing therapy time for patients and infusion centers.^7^ As Dara-SQ became the preferred route of administration, indefinite premedication prior to Dara-SQ continues to be recommended regardless of prior Dara-IV exposure, tolerance of previous doses, or day of therapy within a cycle.^4,8^ Due to the rarity of ARRs after the first dose of Dara-SQ, this study investigated whether premedication may safely be omitted after the first cycle of Dara-SQ to improve the quality of patient care.

## Materials and methods

### Study design and population

This was a single-center, pre- and post-interventional quality improvement study that included patients at least 18 years old and diagnosed with multiple myeloma or AL amyloidosis who received at least one dose of Dara-SQ. Patients were identified using a comprehensive electronic medical record query. Patients were divided into 2 arms based on the presence of premedication omission by physician practice. This project received a formal determination of quality improvement status according to University of Chicago institutional policy; thus, this was not deemed human patient research and was not reviewed by the institutional review board. Data were collected using REDCap (Vanderbilt University).

Per institutional policy, all Dara-SQ naïve patients were monitored for 5 hours after the cycle 1 Day 1 dose of Dara-SQ and for up to 1 hour on subsequent doses if patients were switched from Dara-IV to Dara-SQ. This practice was based on the reduction of ARR incidence after the first dose and real-world clinical practice data.^9^ Premedication was defined as acetaminophen, diphenhydramine, dexamethasone, and montelukast scheduled 1 hour prior to the Dara-SQ dose. Premedication omission was introduced on June 1, 2022 for the post-interventional arm and was defined as the removal of acetaminophen, diphenhydramine, and montelukast prior to Dara-SQ. Dexamethasone was continued as part of the treatment regimen due to its anti-myeloma activity.

### Pre-interventional arm (Arm 1)

Patients in the pre-interventional arm followed the standard of care with indefinite premedication and data on ARRs were collected retrospectively from July 1, 2020 to May 31, 2022.

### Post-interventional arm (Arm 2)

Premedication omission was introduced on June 1, 2022. Patients in the post-interventional arm had premedication omitted starting with cycle 2 day 1. Dexamethasone was also scheduled concurrently with Dara-SQ for all patients in this arm. If an ARR occurred during the first cycle, premedication omission was delayed by physician preference, but no later than starting with cycle 3 day 1. These data were collected prospectively until December 31, 2022.

### Outcomes

The primary endpoint was the incidence of ARRs after premedication omission. Admission-related reactions were defined per Common Terminology Criteria for Adverse Events (CTCAE) v5.0. Reactions were assessed by grade based on supportive care administered during Dara-SQ injection visit and electronic medical record notes from infusion clinic nursing staff surrounding the visit for each Dara-SQ dose. Secondary analyses included overall ARR rate, severity and timing of reactions, reaction management, and post-reaction supportive care.

### Therapy time saved

Cost analysis of therapy time saved was assessed on all Arm 2 post-interventional patients. In the pre-intervention arm, at least 2 hours of ‘infusion chair’ time were scheduled for patients to allow premedication 1 hour prior Dara-SQ and supportive care as needed after administration.

Arm 2 post-interventional patients had only one hour of chair time scheduled for each Dara-SQ administration as dexamethasone was administered concurrently with Dara-SQ specifically in this arm. Therapy time saved between was defined as 1 hour of chair time per each Dara-SQ dose in these post-interventional patients. If additional supportive medications such as anti-emetics, intravenous fluids, electrolyte supplementation, or vaccines were administered at a given visit, the therapy time savings would not be calculated for that Dara-SQ dose. These additional medications (for example, ondansetron) were not given concurrently with Dara-SQ per this study.

### Statistical analysis

Patient characteristics and outcomes between arms were analyzed using STATA software version 17.0 (StataCorp). Wilcoxon rank-sum test was used for continuous variables and the chi-squared test was used to compare binary data between treatment arms.

## Results

A total of 102 patients (63 in Arm 1 and 39 in Arm 2) were included in the final analysis between July 1, 2020 and December 31, 2022. Arm 1 had a higher proportion of prior Dara-IV exposure (52% vs. 10%; *P* value = 0.02) and statistically more patients who transitioned from Dara-IV to Dara-SQ within 3 months (35% vs 0%; *P* value = 0.00003). Arm 2 consisted of more patients with newly diagnosed plasma cell disorders (21% vs 47%; *P* value = 0.03). Complete baseline characteristics are listed in Table 1. For the primary outcome, among the 102 patients across 1479 doses of Dara-SQ, zero ARRs occurred after cycle 1.

**Table 1.** Baseline characteristics.

	Arm 1 *N* = 63	Arm 2 *N* = 39	*P* value
Median age—years (range)	69 (38-86)	70 (51-87)	.58
Median weight, kg (range)	74 (40-131)	74 (51-145)	.36
Median height, cm (range)	170 (145-190.5)	167.6(149-188)	.41
Sex—male (%)	36/63 (56.5)	20/39 (50)	.56
Race (%)			
African American	29 (46)	25 (64)	.08
White	30 (48)	8 (21)	.01
Hispanic	2 (3.2)	3 (7.7)	.92
Asian	1 (1.6)	1 (2.5)	.73
Other	1 (1.6)	2 (5.1)	.30
Prior Dara-IV exposure (%)	33/63 (52)	4/39 (10)	.02
Transition Dara-IV to SQ < 3 months (%)	22/63 (35)	0/39 (0)	.00003
Newly diagnosed (%)	13/63 (21)	16/39 (47)	.03
C1D1 acetaminophen (%)	63/63 (100)	39/39 (100)	1
C1D1 diphenhydramine (%)	62/63 (98)	39/39 (100)	.39
C1D1 montelukast (%)	59/63 (94)	39/39 (100)	.30
C1D1 dexamethasone (%)	63/63 (100)	39/39 (100)	1

Abbreviations: C, cycle; D, day; Dara-IV exposure, any previous intravenous daratumumab given.

Arm 1, pre-interventional; Arm 2, post-interventional.

Secondary analysis showed the overall ARR rate was 3 reactions out of 102 patients (2.9%); 2 reactions occurred in Arm 2, and 1 reaction occurred in Arm 1. Each reaction occurred prior to the premedication omission (Table 2). The median time to the reaction was 4.8 (range 1-5) hours across all reactions and all occurred with cycle 1 day 1 of Dara-SQ. The 3 reactions were grade 2 per CTCAE v5.0. All patients were discharged home from the clinic after the reaction and were able to receive subsequent Dara-SQ doses without further reactions, including the 2 patients in Arm 2 who had premedication omitted no later than cycle 3 day 1 (Table 2).

**Table 2.** Secondary analysis of reactions.

	Arm 1, patient 1	Arm 2, patient 2	Arm 2, patient 3
Day of reaction	C1D1	C1D1	C1D1
Timing (hours)	4.8	1	5
CTCAE Grade	Grade 2	Grade 2	Grade 2
Symptoms	Urticaria, hives, nasal congestion, and sneezing	Chills and rigors	Back pain, rigors, and fever
Emergency medications	Hydrocortisone 100 mg IV	Hydrocortisone 100 mg IV	Hydrocortisone 100 mg IV
	Diphenhydramine 50 mg IV	Diphenhydramine 25 mg IV	Diphenhydramine 25 mg IV
	Tylenol 650 mg PO	Hydromorphone 0.2 mg IV	Morphine 2 mg
Subsequent Dara-SQ doses	No reactions with future doses	Premedication omitted after 6 doses (C2D15) with no further reactions	Premedication omitted after 8 doses (C3D1) with no further reactions

Abbreviations: C, cycle; D, day.

**Figure 1.**
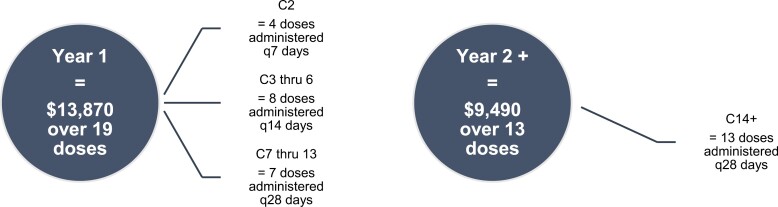
Potential savings per year per patient. Number of doses × $730 USD^8^ = potential savings/patient. Abbreviations: C, cycle; q, every.

Details regarding the treatment regimens and associated therapy time saved are listed in Table 3. Therapy time saved with premedication omission was 194 hours across a total of 202 doses of Dara-SQ given in Arm 2. Extrapolating the National Comprehensive Cancer Network infusion efficiency workgroup study that attributed 1 hour of infusion therapy time is $730 US dollars,^10^ approximately $140 000 US dollars was saved in over a 6-month period (June–December 2022). Assuming standard Dara-SQ dosing, cost savings from chair time reductions on a per patient basis are approximately $13 870 in the first year and $9490 in subsequent years (Figure 1).

**Table 3.** Therapy time saved by regimen.

Regimen	Doses (%)	Hours saved
Total Doses	202	194
Dara-Vd	90 (45%)	90
Dara-Pd	25 (12%)	25
Dara-VRd	24 (12%)	24
Dara-Rd	21 (10%)	21
Dara-maintenance	18 (9%)	18
Dara-Kd	11 (5%)	5
Dara-Dex	8 (4%)	8
Dara-KRd	5 (2%)	3

Abbreviations: V, Velcade; d, Decadron; P, Pomalyst; R, Revlimid; K, Kyprolis.

## Discussion

In this study, we demonstrated the safety of premedication omission after 1 cycle of Dara-SQ in patients who received therapy for plasma cells disorders. Premedication omission saved substantial therapy time for patients and the infusion clinic. Even among patients who experienced an ARR, premedication omission was delayed no later than cycle 3 day 1, which resulted in no further ARRs.

This study has broad application as Dara-SQ is used frequently in the treatment of multiple myeloma and AL amyloidosis and may be given for prolonged durations. A patient with AL amyloidosis who completes the entire 2-year course of Dara-SQ-based therapy as assigned in the ANDROMEDA trial would save 30 hours of infusion time, amounting to approximately $21 900 saved per patient.^2^ A patient with multiple myeloma who completes the 32-cycle course of Dara-SQ-based therapy in the GRIFFIN trial would save 38 hours of infusion time, amounting to $27 740 saved per patient.^11^ Moreover, premedication omission may lead to lower toxicities such as somnolence, headache, or mood alterations as less agents are being administered and increases patient independence allowing safer self-travel to and from appointments. At scale, the time away from infusion clinics for patients and money saved is substantial. This can also substantially increase chair time for other patients who require longer infusion times to finish their therapy, especially in high-volume centers.

A single-center retrospective review found that when premedication was removed after 3-5 doses of Dara-IV, 90-minute rapid infusion of Dara-IV without premedication did not lead to an excess of ARRs compared to those who continued premedication.^12^ The same group investigated the need for premedication prior to Dara-SQ, primarily when switching from Dara-IV, and found no increased risk of ARRs when premedication was omitted.^13^

With respect to Dara-SQ monitoring, a recent single-center experience found it safe to eliminate monitoring even as early as cycle 1 day 1.^14^ A secondary outcome observed that the omission of acetaminophen and diphenhydramine following the third dose of Dara-SQ resulted in an all-grade ARR rate of 4.7% (0% grade 3 or higher), similar to this study.^14^ Another recent retrospective analysis showed that there was no difference in ARRs when premedication was de-escalated after cycle 1 between arms with all ARRs also occurring in cycle 1, in line with our study.^15^ Although, some patients in the post-implementation arm did receive some form of premedication (16.7%) with cycle 2 onwards.^15^

The limitations of this study include its retrospective single-center design, along with an imbalance between certain patient characteristics between the groups. Dexamethasone was continued and given concurrently with Dara-SQ in this study and may have contributed to the low rate of ARRs in this study. It is notable that the arms were imbalanced; the pre-intervention arm (Arm 1) had a higher percentage of patients with prior Dara-IV exposure and the post-intervention arm (Arm 2) had a higher percentage of patients with newly diagnosed plasma cell disorders. However, if anything, this should have accounted for potentially more ARRs in Arm 2 because there were more Dara-SQ naïve patients which likely have an increased risk for ARRs. Another limitation was potential undocumented information outside of the standardized ARR documentation. This likely did not miss any ARRs given that infusion nurses are instructed to inquire about previous reactions prior to each dose of Dara-SQ.

## Conclusion

Findings from this study showed that ARRs due to Dara-SQ were rare, mild in course, and occurred early. To our knowledge, this study was the largest quality improvement initiative with premedication omission among Dara-SQ treatment regimens. The omission of premedication was safe, feasible, and carried substantial time and cost savings for patients and infusion centers. This therapy adjustment should be considered for all Dara-SQ treatment regimens.

## Data Availability

The data underlying this article will be shared on reasonable request to the corresponding author

## Prior presentation

Early results in poster format presented in part at the 2023 HOPA annual meeting, in person, March 3, 2023. Presented in poster format in part at the 2023 ASCO annual meeting, in person, June 3, 2023.
